# Pseudoesotropia in Chinese Children: A Triphasic Development of the Interepicanthal Folds Distance-to-Interpupillary Distance Ratio and Its Changing Perception

**DOI:** 10.1007/s00266-018-1298-4

**Published:** 2019-01-09

**Authors:** Nan Wei, Xuehan Qian, Hua Bi, Xiaoli Qi, Hongyu Lu, Lirong Wei, Xue Li, Fengyuan Sun, Bin Zhang

**Affiliations:** 1 Department of Pediatric Ophthalmology and Strabismus, Tianjin Medical University Eye Hospital, Tianjin, 300384 China; 2 College of Optometry, Nova Southeastern University, Fort Lauderdale, FL 33314 USA; 3 Department of Children Eye Care, Maternity and Child Care Center of Qinhaungdao, Qinhuangdao, 066000 Hebei China; 4 Department of Ophthalmology, Tianjin Beichen women and Children’s Health Center, Tianjin, 300000 China; 5 Department of Ocular Plastic and Orbital Disease, Tianjin Medical University Eye Hospital, Tianjin, 300384 China

**Keywords:** EFDPD ratio, Epicanthus, Chinese children, Pseudoesotropia, Perception

## Abstract

**Purpose:**

To delineate the development of the interepicanthal fold distance (IEFD) to interpupillary distance (IPD) in Chinese children, and to quantify how their ratio (EFDPD ratio) affects parent’s judgment on whether a child’s two eyes appear misaligned.

**Methods:**

The values of IPD and IEFD were measured in 750 children, aged between 3 and 17 years. The developmental trend of EFDPD ratio was established. Two hundred parents were shown a series of pictures of children with varying EFDPD ratios and asked to judge whether the child in each picture demonstrated misaligned eyes. Based on the parent’s responses, psychometric functional associations with EFDPD ratios were established.

**Results:**

The EFDPD ratios were significantly higher (0.63 ± 0.027) and showed little change among children from 3 to 6 years of age (*p* = 0.704). During the age of seven to 12 years, however, the EFDPD ratio significantly decreased (*p* < 0.001) before stabilizing at 0.59 ± 0.023 by the ages of 13 to 17 years (*p* = 0.376). Children with EFDPD ratios > 0.65 were more likely to be perceived as strabismic by the parents, while children with an EFDPD ratio < 0.55 were rarely perceived as so. As many as 30% of the children aged between 3 and 6 years demonstrated EFDPD ratios > 0.65, and this number reduced to 5% by the age of 12 years.

**Conclusions:**

The development of the EFDPD ratio in Chinese children shows a triphasic pattern, with a large value before the age of 6 years, a quick drop between 7 and 12 years, and little change after 13 years of age. As the EFDPD ratio declines, fewer children appear as strabismic.

**Level of Evidence IV:**

This journal requires that authors assign a level of evidence to each article. For a full description of these Evidence-Based Medicine ratings, please refer to the Table of Contents or the online Instructions to Authors www.springer.com/00266

## Introduction

In comparison with Caucasian children, Chinese children have a more prominent medial epicanthus and a flatter nasal bridge [1, 2]. This may lead to the appearance of the eyes being crossed, particularly with a head turn or during a lateral gaze. It is often noted that concerned parents bring in their children, whose eyes are perfectly aligned, to the pediatric ophthalmologists for consultation. In infants, it accounts for up to ten percents of outpatient visits [3, 4]. During the consultation, the orthotropic appearance can be demonstrated to the parents by pinching slightly the child’s nasal bridge and revealing the nasal sclera [5–7], and the concept of false appearance of squints can be explained by placing an artificial broad nose between two straight eyes sketched on a card board [8].

Textbooks of ophthalmology usually state that those children’s eyes will outgrow this condition [6, 9, 10]. However, the descriptions are often brief. Moreover, few, if any, specific reference values are provided to answer the questions frequently asked during the consultation. For example, parents of infants or young children are anxious to know whether and/or at what age the child’s eyes will appear normal [4]. For school age children who still appear as squinting and are sensitive to the looks, their parents are concerned more about the best time for a cosmetic surgery since the child’s facial structures are still in development [11]. For an adult with pseudoesotropia who comes for a cosmetic surgery, the doctor needs to decide to what extent the correction should be carried out [11].

To provide the reference values for better clinical decision-making, quantitative investigations are necessary in the following two aspects. The first key aspect is mapping out the normal development of structures surrounding the eyes and nasal bridge, which are changing constantly until the teenage years [12–17]. When asked what made them believe that their child’s eyes are turned inward, parents often present the notion that the white area between the inner corner of the eye (the medial canthus) and the edge of the black area (the pupil) seems to be too small. Therefore, the first aim of the present study is to quantify the developmental curve for the ratio of the interepicanthal fold distance (IEFD) and interpupillary distance (IPD) (EFDPD ratio) in children aged between 3 and 17 years of age.

The second key aspect is the degree of the parent’s reaction to such an anatomical change. The relationship between the EFDPD ratio and the parent’s perception of this anatomical feature may not be a linear one. There might exist a threshold below which people tend to more likely think that the two eyes are aligned, and above which people tend to more likely think that the two eyes are not aligned. Therefore, the second aim of the present study was to map out this psychological response curve to the EFDPD ratio and to identify this threshold from the response curve. The combination of the findings from these two angles of view may provide valuable references for clinical consultation and decision-making.

## Methods

### Subjects

From March 2016 to May 2017, a total of 17,588 children, between 3 and 17 years old, were screened (demographics, Table 1). All procedures performed in this study were in accordance with the ethical standards of the institutional research committee of Tianjin Medical University and with the 1964 Declaration of Helsinki and its later amendments or comparable ethical standards. Written informed consent was obtained from the parents of the children involved prior to any testing. Additional informed consent was obtained from the parents of the children for whom identifying information is included in this article.

**Table 1:** Number of children screened in each age-group

Age (years)	3	4	5	6	7	8	9	10	11	12	13	14	15	16	17
*N*	236	1871	2362	2070	937	1067	1096	1049	1015	1047	890	1018	986	1017	927

### Screening Method and EFDPD Ratio Calculation

**Fig. 1:**
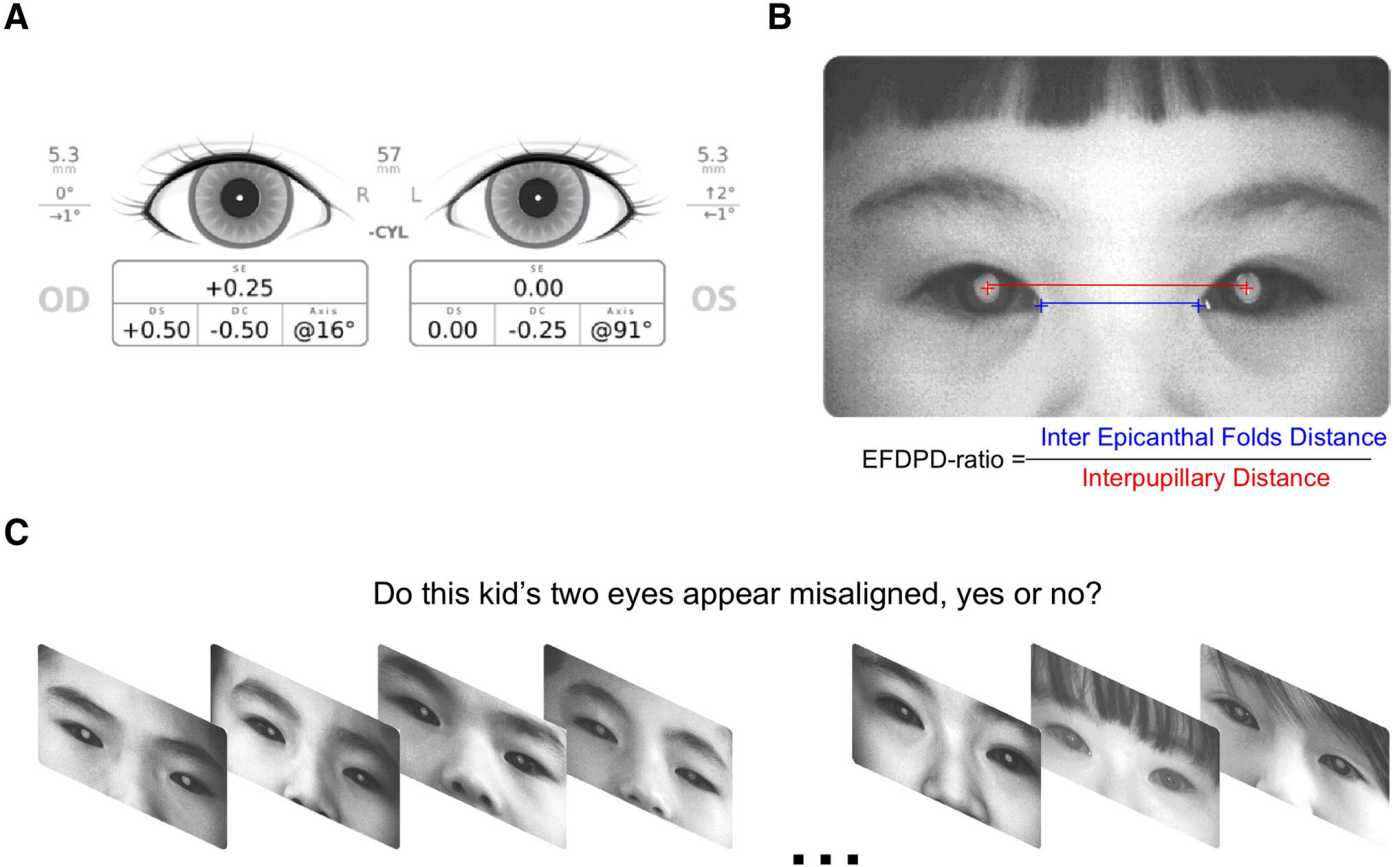
Experimental methods. **a** An exemplary SPOT™ Vision Screener screen data. **b** The calculation of the EFDPD ratio. **c** The random stimuli method used to measure parents’ perception of eyes with varying EFDPD ratios. For each photograph, parents answered ‘yes’ or ‘no’ to the question whether the child’s two eyes appear misaligned

Participant screening was performed via the SPOT™ Vision Screener, a handheld infrared photoscreener (Welch Allyn, Skaneateles Falls, NY, USA). During the screening, participants were asked to gaze at the device with both eyes. As such, once an image of the red reflex was successfully acquired, the device was able to automatically report the non-cycloplegic refractive status, pupil size, IPD, and gaze deviation within 2 s (Fig. 1a). The SPOT™ Vision Screener is programmed to automatically flag a referral for a complete eye examination if it detects significant refractive error [e.g., myopia < − 1.0 diopter (D), hyperopia > 1.0 D, astigmatism > 1.0 D]; anisometropia (> 1.0 D); anisocoria; or strabismus [18]. The data of subjects with these characteristics were subsequently not included in further analysis. Potential participants were also excluded from further tests if they demonstrated craniofacial abnormalities, ptosis, or other diseases that may affect the eye appearance. Following screening, a total of 2,788 participants were deemed eligible for further analysis on IPD. Because it is impractical to extract the data for the medial canthus from a sample size as large as that in the current study, to facilitate the data analysis, only a portion of the subjects was further chosen from the study population for further evaluation: Specifically, for each age, we selected 25 boys and 25 girls whose IPDs were close to the group median for IEFD analysis.

Images captured by the SPOT™ Vision Screener were imported into ImageJ, an image-analysis software available in the public domain (National Institutes of Health, Bethesda, MD, USA). An examiner manually clicked on the two points representing the pupil centers, as well as the corners of the left and right medial canthi. ImageJ can also be used to export the x and y pixel locations of those identified points. Since the SPOT™ Vision Screener reports the IPD values in millimeters (mm), the IEFD can also be calculated in mm. The ratio between the IEFD and IPD was calculated for each participant and recorded as the EFDPD ratio (Fig. 1b).

### Psychophysical Study to Establish a Response Curve for Varying EFDPD Ratios

To study how parent perceptions of whether a child demonstrated misaligned eyes or not changed with varying EFDPD ratios, a total of 200 parents who had brought their children to visit the clinic for routine refractions were recruited to join the study. Written informed consent was obtained from the parents before initiation of the study. Accordingly, using a random sequence, each parent was shown a series of 35 images that contained the seven EFDPD ratios representing the 5th, 15th, 30th, 50th, 70th, 85th, and 95th percentiles of the population. All children depicted in the images were healthy, had no strabismus, and were not related to any of the parents. For each picture, parents were required to provide an answer of yes or no as to whether the eyes of the child in the picture appeared to be misaligned (Fig. 1c). All parent responses were recorded and separated into the groups of the different EFDPD ratios. The percentage of answering ‘yes’ in each bin was computed and plotted as a function of varying EFDPD ratios.

### Statistics and Mathematical Analysis

All statistical analyses were performed using SPSS statistical software version 20 (IBM, Armonk, NY, USA). Kolmogorov–Smirnov tests were used to assess whether the distribution of IEFD, IPD, and EFDPD ratios was normal. To compare whether the values of IEFD, IPD, and EFDPD ratios were different across age-groups, we used one-way analysis of variance. Two-tailed statistical significance was determined using an alpha level at 0.05. The developmental trends of the IPD, IEFD, and EFDPD ratios were first fitted into polynomial functions to revel the general trends, and then EFDPD ratio data were fitted into a piece-wise linear regression model for further analysis. The association between parent’s perceptions of misaligned eyes and varying EFDPD ratios was fitted into a cumulative normal curve. The relationship between age and the percentage of children with EFDPD ratios greater than 0.65 was also fitted into a reverse cumulative normal curve.

## Results

### Interpupillary Distance (IPD), Interepicanthal Folds Distance (IEFD), and EFDPD Ratios

The values of IPD, IEFD, and EFDPD ratios for normal children between 3 and 17 years of age are presented in table (Table 2).

**Table 2:** IPD, IEFD, and EFDPD ratios for normal children aged between 3 and 17 years

Age (years)	IPD		IEFD		EFDPD ratio	
	*N*	Value	*N*	Value	*N*	Value
3	189	52.27 ± 3.15	50	33.09 ± 1.67	50	0.6305 ± 0.0266
4	200	53.13 ± 3.10	50	33.69 ± 1.74	50	0.6326 ± 0.0288
5	200	55.42 ± 3.01	50	34.77 ± 1.70	50	0.6300 ± 0.0272
6	200	56.66 ± 3.28	50	35.96 ± 2.12	50	0.6328 ± 0.0341
7	200	57.41 ± 3.03	50	35.53 ± 2.06	50	0.6227 ± 0.0323
8	200	58.70 ± 3.13	50	36.13 ± 2.24	50	0.6157 ± 0.0321
9	200	58.93 ± 3.06	50	35.87 ± 2.15	50	0.6132 ± 0.0321
10	200	60.14 ± 3.23	50	36.12 ± 1.82	50	0.6049 ± 0.0296
11	200	61.48 ± 3.35	50	37.18 ± 1.85	50	0.6077 ± 0.0269
12	200	63.01 ± 3.45	50	37.87 ± 2.22	50	0.6020 ± 0.0305
13	200	63.75 ± 3.51	50	37.81 ± 1.93	50	0.5933 ± 0.0272
14	200	64.42 ± 3.51	50	38.20 ± 2.09	50	0.5955 ± 0.0281
15	189	64.95 ± 3.47	50	38.42 ± 2.13	50	0.5909 ± 0.0301
16	100	65.85 ± 3.80	50	39.19 ± 2.48	50	0.5949 ± 0.0327
17	99	65.53 ± 3.20	50	38.61 ± 1.91	50	0.5880 ± 0.0241

**Fig. 2:**
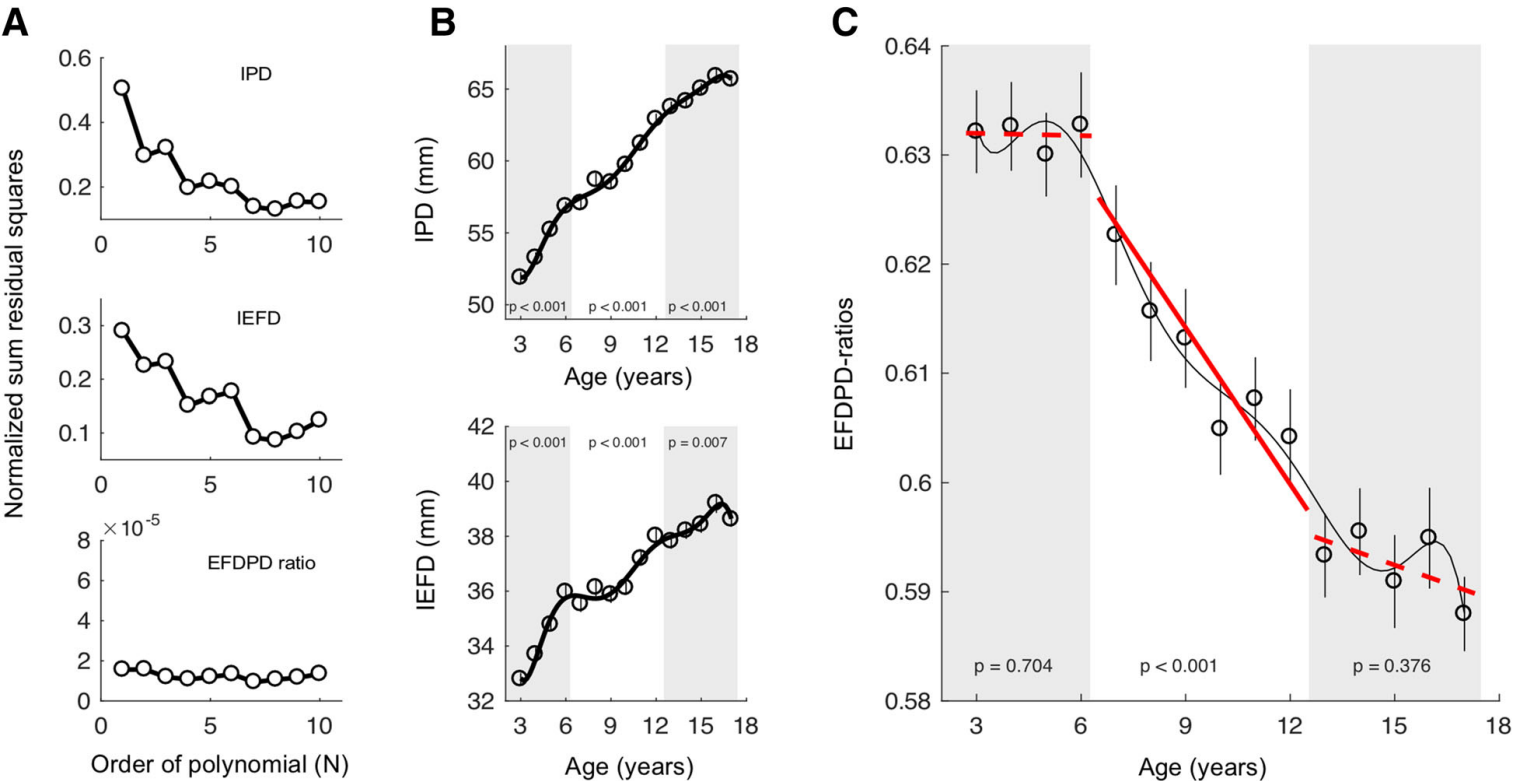
Development of the IPD, IEFD, and EFDPD ratios in children aged between 3 and 17 years. **a** Fitting errors versus the order of polynomial for IPD (top panel), IEFD (middle panel), and EFDPD ratios (bottom panel). **b** Polynomial functions of the seventh order were fitted for the development of IPD (top panel) and IEFD (bottom panel). **c** Developmental trend of EFDPD ratio was fitted into a polynomial function of the seventh order (thin black line) and piece-wise linear regression (red lines). Solid red line indicated a slope significantly different from zero to dash line indicated a slope not significantly different from zero

The development of IPD is not linear. We fitted polynomial functions from the order of one to the order of 11 and plotted the normalized sum of residual squares (Fig. 2a, top panel). A polynomial of the seventh order was the best fit with the smallest amount of fitting error, which was shown in the top panel of Fig. 2b. From the curve, the development of IPD could be roughly divided into three stages, from 3 to 6 years of age, from 7 to 12 years of age, and from 13 to 17 years of age, with the developmental speeds gradually slowing down. In each stage, the IPD significantly increases (*p* < 0.01 for each phase, see Fig. 2b, top panel). A similar trend was found for the development of IEFD (Fig. 2a, middle panel and Fig. 2b, bottom panel).

The development trends of IPD and IEFD were approximately parallel to each other for both the 3 to 6 years stage and the stage between 13 and 17 years, but became diverged for the stage from 7 to 12 years. Due to the variations in the IPD and IEFD values, it seemed arbitrary to divide the development into three stages. By computing the EFDPD ratio, the variations specific to IPD or IEFD canceled each other out, and a clear triphasic pattern was revealed. The EFDPD ratios were high and showed little change at the early stage (*p* = 0.704 for age of three years to six years), and were maintained at a low level for the late stage (*p* = 0.376 for the age of 13 years to 17 years). However, EFDPD ratios greatly decreased during the middle stage (*p *< 0.001, from 7 to 12 years of age). Since polynomial fitting was not better than linear fitting (Fig. 2a, bottom panel), this rapid decline was fitted into a linear line (red line, Fig. 2c).

### Response Function to EFDPD Ratios

**Fig. 3:**
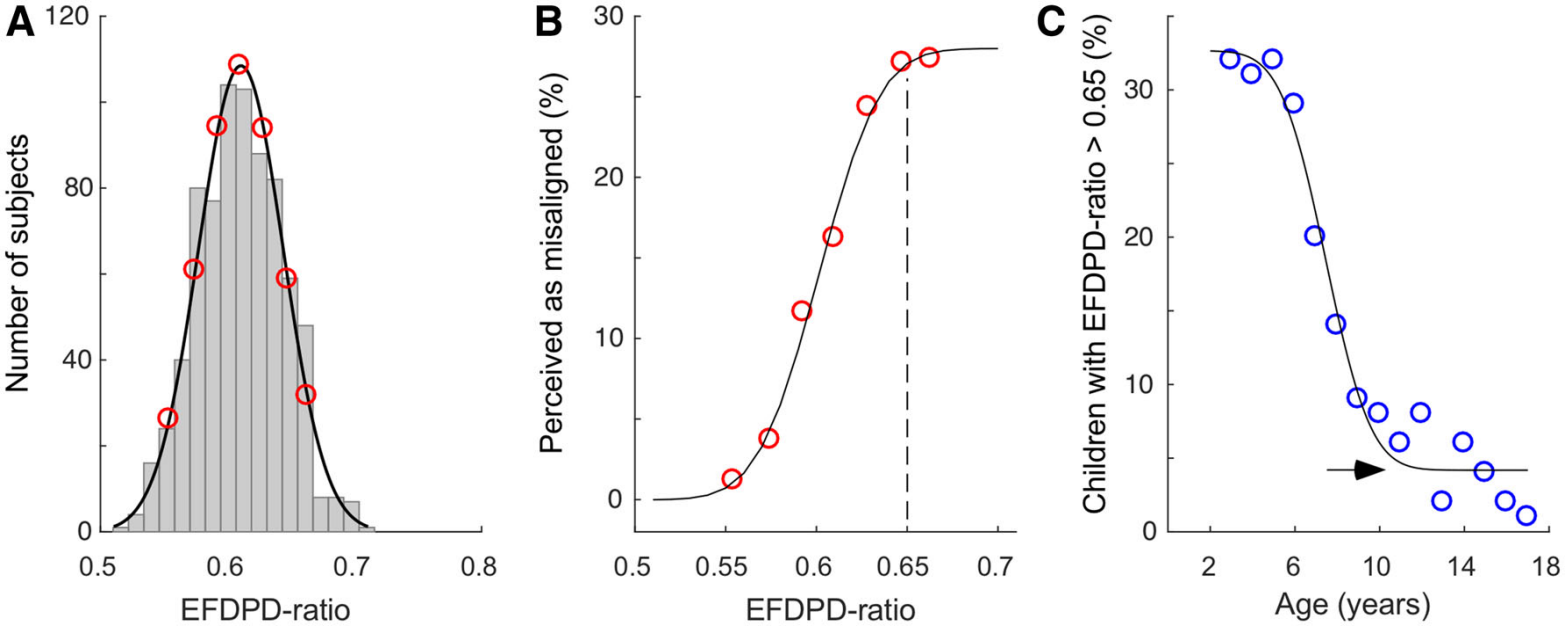
Relationship between parental perception of ocular misalignment in children and EFDPD ratios. **a** Distribution of EFDPD ratios. **b** The percentage of children perceived to be misaligned eyes, based on the response from 200 parents, increased with EFDPD ratio. **c** The percentage of children with EFDPD ratios larger than 0.65 decreased with age

The distribution of the EFDPD ratios for the 750 pediatric participants fits a normal distribution pattern (Fig. 3a). With this distribution, the EFDPD ratios representing the 5th, 15th, 30th, 50th, 70th, 85th, and 95th percentiles were used to test the parent’s perceptions on varying EFDPD ratios (red dots in Fig. 3a). A cumulative normal curve was fitted into the data points to summarize the relationship between perception and EFDPD ratios. When an image had an EFDPD ratio greater than 0.65, roughly 30% of the tested parents thought the pictured child was strabismic. When the EFDPD ratio was in the range of 0.55 to 0.65, the percentage of parents who perceived the existence of strabismus rapidly declined. Lastly, in images demonstrating an EFDPD ratio of less than 0.55, it was highly unlikely that the child would be perceived as demonstrating strabismus (Fig. 3b).

Since children who demonstrated EFDPD ratios greater than 0.65 were likely to be identified by the parents as ‘abnormal,’ we counted the percentage of children demonstrating EFDPD ratios greater than 0.65 for each age (Fig. 3c). Notably, up to 30% of the children aged between 3 and 6 years demonstrated EFDPD ratios of greater than 0.65. However, this number rapidly declined during the subsequent years. By the age of 12 years, this number was stabilized at the chance level.

## Discussion

### Choice of the EFDPD Ratio

Previous studies have reported the absolute values of the IPD and/or IEFD during childhood development [13, 16, 19]. Although these data are valuable in our understanding of early childhood development, this traditional approach is limited because both IPD and IEFD demonstrated high levels of variance among heterogeneous individuals [15, 20–22]. As such, in the present study, we suggest that there is value in using the ratio of the two variables as an index [23]. By taking the ratio of IEFD and IPD, two important advantages can be achieved. First, since both IPD and IEFD are obtained from the same individual, intra-individual variance tends to cancel out, leaving the ratio to reflect the true population mean better (See Fig. 2b). Second, the overall range, or scales, of individual measurements is broader for both IPD and IEFD. As such, by combining those two parameters into a single ratio, the range of the values is limited to between 0 and 1. Thus, the interpretation of the relative contribution of IPD and IEFD was made much easier.

### The Triphasic Development of the EFDPD Ratio

The three stages of development of the EFDPD ratio can be explained by the differences in the developmental speed between IPD and IEFD. Before 6 years of age, both IEFD and IPD develop rapidly and at nearly the same speed, resulting in an invariable EFDPD ratio. However, from 7 to 12 years of age, the development of both IEFD and IPD begins to slow down, but with the deceleration more pronounced in IEFD, leading to a decreased EFDPD ratio during this developmental period. Lastly, after an individual reaches 13 years of age, IEFD and IPD develop at similar speeds again and the EFDPD ratio flattens out once more.

The development of the IPD, IEFD, and EFDPD ratios should not be studied in isolation. Rather, they should be considered as a part of the development of the craniofacial features, which is a complex process that involves many components, including the bony structure, the sinuses, and the soft tissues such as skin and muscle, with each one developing in a different pattern and at different speeds [24–26]. The quick decline of the EFDPD ratio during the 7 years to 12 years of age period is caused by the relatively faster growth rate of IIPD (see Fig. 2b). The expanding of the IIPD may rely on the enlargement of the bony structures, orbits in particular, to allow for sufficient space. This is supported by the findings that the orbit experiences a growth spurt between 7 and 12 years of age [27]. It is also congruent with the finding that a rapid expansion of the sinuses occurs after 7 years of age [28].

### Chinese Versus Western Children

In comparison with Caucasian populations, it is well recognized that the epicanthus is more prominent in eastern Asian populations [1, 2]. Therefore, it is relevant to compare the values of the EFDPD ratio directly between these two populations. To date, there have been no direct reports on the development of this ratio. As such, in the present study, we took the developmental values of IPDs and IEFDs from two different studies on Western Children and computed the EFDPD ratios [29, 30]. Although one should interpret these data with caution, our observations provide some insight into this particular issue. As compared with Chinese children, the overall EFDPD ratio of the Western children, between birth and 18 years of age, is significantly lower (i.e., in the range of 0.53 to 0.56), close to the number of 0.5 reported in Western adults [31]. Importantly, this is the range least likely to cause a parent to perceive misaligned eyes in a child. The gradual reduction in the EFDPD ratio demonstrated by Western children is not as dramatic as compared with that in Chinese children.

### Clinical Significance of the EFDPD Ratio

To the best of our knowledge, this is the first study that shows the relationship between parental perception of ocular misalignment and EFDPD ratios. We have demonstrated that not only are physiological changes in the children impactful, but also that parent perceptions play an important role. Thus, we provide direct evidence to suggest that ignoring either the child or parent aspect would present an incomplete picture of the need to potentially attend to pseudoesotropia.

The findings from the present study may help to address the three questions aforementioned. First, the EFDPD ratio declines quickly between the ages of 7 and 12 years, and the percentage of the children with EFDPD ratios greater than 0.65 also rapidly reduced to the chance level by 12 years of age. Therefore, the parents of infants and young children should expect the appearances of their children to improve significantly by the age of 7 years to 12 years. Second, the EFDPD ratio stabilizes around the age of 13 years and the percentage of children with EFDPD ratios greater than 0.65 decreases to a very low level at around 12 years of age. Therefore, it seems 12 years to 13 years of age would be the earliest time for surgical consideration if the child and parents do not want to wait longer. This should only be used as general guidance, and the decision for a patient should take into consideration other individual specific information, such as whether the patient has epiblepharon [32]. Third, for those adults who have already passed the developmental period, surgical procedures would be aimed at reducing the IEFD values so as to bring the EFDPD ratio close to 0.55. Certainly, planning a cosmetic surgery involves many other factors, and the EFDPD ratio only provides a reference value from one aspect. In the future, we will study how patient and parental acceptance and satisfaction with post-surgical outcomes correlate with EFDPD ratio reduction. The present and future quantitative analysis of EFDPD ratio dynamics is expected to provide valuable guidance in helping concerned family members make the best decisions for their children.

## Conclusions

Chinese children have large ratios before the age of 6 years. Between 7 and 12 years of age, the EFDPD ratios drop quickly and then stabilize by 13 years of age. Importantly, children demonstrating EFDPD ratios of more than 0.65 are more likely to be perceived by their parents as having misaligned eyes.
